# Helium Bubbles and Blistering in a Nanolayered Metal/Hydride Composite

**DOI:** 10.3390/ma14185393

**Published:** 2021-09-18

**Authors:** Caitlin A. Taylor, Eric Lang, Paul G. Kotula, Ronald Goeke, Clark S. Snow, Yongqiang Wang, Khalid Hattar

**Affiliations:** 1 Materials Science and Technology Division, Los Alamos National Laboratory, Los Alamos, NM 87545, USA; caitlin@lanl.gov (C.A.T.); yqwang@lanl.gov (Y.W.); 2 Component Science, Engineering, and Production Center, Sandia National Laboratories, Albuquerque, NM 87185, USA; rsgoeke@sandia.gov (R.G.); cssnow@sandia.gov (C.S.S.); 3 Material, Physical, and Chemical Sciences Center, Sandia National Laboratories, Albuquerque, NM 87185, USA; ejlang@sandia.gov (E.L.); pgkotul@sandia.gov (P.G.K.)

**Keywords:** tritium storage, functional materials, nanolayers, multilayers, helium implantation, helium bubbles, metal hydrides, radiation damage, nuclear materials

## Abstract

Helium is insoluble in most metals and precipitates out to form nanoscale bubbles when the concentration is greater than 1 at.%, which can alter the material properties. Introducing controlled defects such as multilayer interfaces may offer some level of helium bubble management. This study investigates the effects of multilayered composites on helium behavior in ion-implanted, multilayered ErD_2_/Mo thin film composites. Following in-situ and ex-situ helium implantation, scanning and transmission electron microscopy showed the development of spherical helium bubbles within the matrix, but primarily at the layer interfaces. Bubble linkage and surface blistering is observed after high fluence ex-situ helium implantation. These results show the ability of metallic multilayers to alter helium bubble distributions even in the presence of a hydride layer, increasing the lifetime of materials in helium environments.

## 1. Introduction

Metal hydrides can store large volumes of hydrogen to relatively high temperatures, making them desirable for a variety of applications, including: hydrogen transportation and storage, neutron moderators in fission energy systems, radiation shielding, and tritium storage for fusion energy. Rare earth hydrides, such as ErH_x_, typically exhibit better hydrogen retention at elevated temperatures than transition metals [1]. Little information exists on radiation effects in metal hydrides, even though they are under consideration for a variety of nuclear applications. In tritium storage applications, tritium β-decays to ^3^He with a half-life of 12.3 years, resulting in substantial rapid build-up of helium inside the hydride lattice. Helium accumulation occurs in many materials exposed to radiation environments, including structural materials in fission reactors and nuclear waste form materials, and often results in degradation of material properties. Helium is insoluble in most materials and precipitates out of the matrix to form clusters and bubbles when the helium concentration is sufficiently high [2,3,4]. Helium agglomerates usually assume an over-pressurized spherical morphology in metal systems but can link to form platelets or other morphologies in some cases [5,6,7,8]. The addition of He often causes mechanical property degradation in materials [2,9,10]. Bubble linkage is of particular concern to the mechanical integrity of the material because they can more easily link and evolve into cracks than individual nanometer-sized spherical bubbles [11,12,13,14,15]. These cracks can evolve into blisters if the material plastically deforms before failing. Blistering is a process that occurs when sub-surface gas-filled cavities reach sufficient pressure to deform the material into micron-sized, dome-shaped structures at the surface [16,17,18,19,20,21,22,23]. Blisters have been observed on the surface of implanted Er and Er hydride [11,12,13]. Blisters can burst if the pressure becomes high enough, causing spallation at the surface [21,23].

Helium is known to trap in areas with large free volumes, such as in radiation-induced vacancies or clusters and at interfaces such as grain boundaries [24,25,26,27,28,29]. Several authors have previously utilized multilayered structures, which contain a high density of incoherent interfaces, to trap and retain large quantities of He in the multilayer structure, thereby weakening the ability to form blisters and enhancing radiation tolerance [27,30]. Helium bubbles have been shown to preferentially nucleate along nanolayer interfaces in several systems, including: Cu/Nb [26,27,31,32,33,34,35,36,37], Cu/Fe [38], Cu/V [39,40,41], Al/Nb [42], Cu/W [43,44,45], Fe/TiO_2_ [46], Fe/W [47], and V/Ag [48]. Molecular dynamics simulations of the Cu/Nb and Cu/V structures have shown strong He binding to face-centered cubic (fcc)/body-centered cubic (bcc) multilayer interfaces [26,27,30]. In all cases, the layer thickness must be sufficiently small for He to diffuse to the interfaces without being trapped by other defects along the way. To form these structures, two immiscible metals must be joined to form alternating layers that will not mix to form a single phase. Most of the aforementioned studies accomplish this using thin-film deposition, but some work uses accumulative roll bonding [31,38]. In contrast to the other multilayer systems, in this work, we chose to layer ErD_2_ with Mo through thin-film deposition and a subsequent hydriding process. Er is a hexagonal metal that transforms to ErD_2_ in the fcc lattice configuration during hydriding, and Mo is a bcc structure that is immiscible with Er up to 1477 °C. ErD_2_ acts as a surrogate for radioactive ErT_2_ in this work. The interfaces are, therefore, fcc/bcc in the nanolayered hydride composite. The ErD_2_/Mo nanolayered composites were implanted with He using an ion accelerator to simulate the evolution of He aging. Advanced electron microscopy techniques, including scanning transmission electron microscopy, energy dispersive X-ray spectroscopy analysis, automated crystallographic orientation mapping, and in-situ helium implantation, were used to characterize bubble nucleation and growth.

## 2. Materials and Methods

### 2.1. Nanolayered Composite Preparation

A multilayered structure of alternating ErD_2_ and Mo layers was prepared by first depositing alternating layers of Er and Mo. Six sets of Er/Mo layers of approximately 20 nm in thickness were deposited via electron-beam evaporation at a rate of 0.5 nm/s on top of the 150 nm W layer sputter deposited on a Si substrate, and capped with an additional Mo layer to slow oxidation at the surface. Samples were diced into 1 × 1 cm squares then heated in a vacuum system and exposed to deuterium to form the hydride. This hydriding process results in alternating ErD_2_ and Mo layers, as Mo does not form a hydride. Because Er and Mo are immiscible up to 1477 °C, intermetallic phases do not form during the nanolayer deposition or during the hydriding. The chamber pressure was 5 × 10^−7^ or less Torr prior to deposition and hydriding. Although hydrogen diffusion through Mo is slow, hydriding is expected to occur through the cut edges of the diced samples. Achievement of the ErD_2_ phase was verified by x-ray diffraction (XRD, (D8 Discover, Bruker, Billerica, MA, USA), which also indicated the presence of Er_2_O_3_ and Mo in the samples (See Figure S1 in Supplementary Files). No other oxide or intermetallic phases were identified in the XRD analysis.

### 2.2. In-Situ Ion Implantation

In-situ transmission electron microscopy (TEM, JEOL 22100, JEOL Ltd., Tokyo, Japan).^4^He implantations were performed using the In-situ Ion Irradiation TEM (I^3^TEM) at Sandia National Laboratories. Implantations were conducted using 10 keV He^+^ produced by the Colutron accelerator [49,50]. This ion energy deposits ^4^He with the concentration peak near the center of the sample. The implantations were undertaken in low magnification mode to produce a wider ion beam inside the TEM. The electron beam was blanked during implantations to limit electron beam effects. Implantations were conducted at a tilt of 30° toward the ion beam. Imaging was performed by temporarily blocking the ion beam, tilting back to 0°, and inserting the objective aperture. The multilayer interfaces could not be imaged at 30° tilt because they became smeared upon tilting. Images were collected after reaching several fluences once He bubbles were observed. Bubbles were identified using the Fresnel contrast method in bright-field (BF) TEM, where bubbles appear light in under-focus conditions and dark in the same location in over-focus conditions. Images were collected at ±518 nm and ±1036 nm defocus. The beam spot was burned into tape and had a measured area of 72 mm^2^. The beam area measurement was approximate due to the non-uniform nature of the ion beam profile, which was not rastered. The electron beam was also burned into the same piece of tape to verify that the electron and ion beams intersected, thus indicating that the ion beam was hitting the sample during the in-situ implantation. The ion beam current was approximately 2 µA, as measured every 15 min by the Faraday cup directly upstream of the TEM, and remained stable throughout the implantation. The implantation was performed at nominally room temperature. Overall beam heating from similar beams was measured previously and was considered negligible. In-situ ion irradiation samples were prepared using an FEI Scios focused ion beam (FIB) system equipped with a Ga ion source at 30 keV.

### 2.3. Ex-Situ Ion Implantation 

Ex-situ ^4^He implantation conditions were determined using Monte-Carlo simulations in the Stopping and Range of Ions in Matter (SRIM, 2013 version) software to simulate the ion implantation depth as a function of ion beam energy [51]. Implantations were performed on a 200 kV Varian Ion Implanter at Ion Beam Materials Laboratory at Los Alamos National Laboratory with 30 keV ^4^He^+^ ions under room temperature to deposit the He concentration peak in the center of the nanolayered composite structure. The implantation fluences varied from 7 × 10^16^, 1 × 10^17^, 1.5 × 10^17^, and 2.5 × 10^17^ ions/cm^2^ with a beam flux of approximately 1.5 × 10^13^ ions/cm^2^/s. The samples were tilted 5 degrees in reference to the beam to avoid any channeling effects. The beam heating effect was found to be negligible due to the large steel cube holder used.

### 2.4. Microstructure Characterization 

Samples implanted ex-situ were characterized using a combination of scanning transmission electron microscopy (STEM) high angle annular dark field (HAADF) imaging, and energy dispersive X-ray spectroscopy (EXDS). In samples containing nanoscale grains, HAADF imaging provides better contrast for He bubble identification than bright-field (BF) TEM imaging techniques. STEM was performed with an FEI Company (Hillsboro, OR, USA) Titan G2 80-200 operated at 200 kV and equipped with four silicon-drift X-ray detectors for EDXS. EDXS (SuperX, FEI Company, Hillsboro, OR, USA) was used to identify the ErD_2_ and Mo layers. Samples were characterized before and after hydriding using Nanomegas ASTAR precession-assisted automated crystallographic orientation mapping (ACOM) in a JEOL 2100 TEM (JEOL Ltd., Tokyo, Japan). ACOM parameters included a 2 nm step size and a precession angle of 0.1°. Analysis was performed using Nanomegas software (version). CrystalMaker software was used to produce images of the crystal structures and to verify electron diffraction patterns. 

Helium bubble analysis was performed with the “Analyze Particles” function in ImageJ [52]. Prior to analysis of bubble size and density, the TEM micrographs were filtered to aid in the visualization of bubbles. All micrographs were filtered with the same routine prior to analysis, including: (a) FFT bandpass filter, (b) thresholding, (c) utilizing the “Normalize Local Contrast” integral image filter, (d) salt-and-pepper noise addition, (e) de-speckle noise filtering, (f) manually filling in pixels within bubbles that appeared to be improperly thresholded, and (g) analyzing the particles. Bubble sizes were filtered to be greater than 1 nm^2^. The wide range of characterization techniques permitted a detailed characterization of the first known study of accelerated aging of ErD_2_/Mo multilayers.

## 3. Results

### 3.1. Nanolayer Structure before and after Hydriding

**Figure 1:**
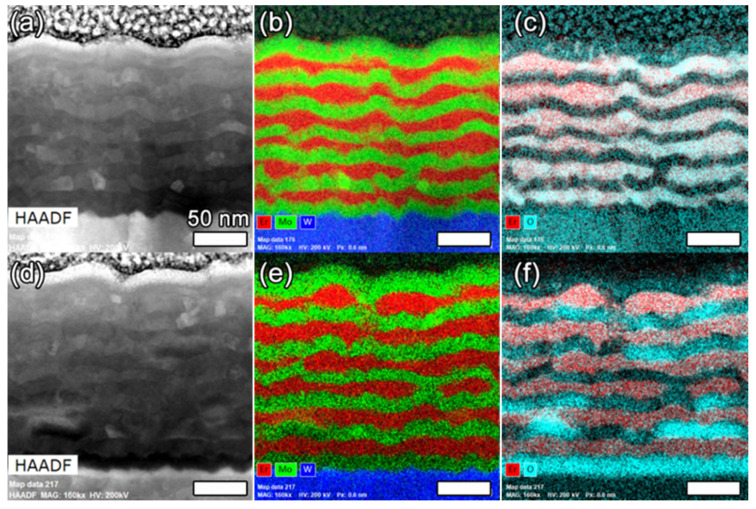
EDXS maps showing the nanolayers (**a**–**c**) before and (**d**–**f**) after hydriding. Overlays of the O and Er EDXS maps in (**c**) and (**f**) show the correspondence between these elements, which are indicative of Er oxide formation supporting the XRD spectra and show the presence of Er_2_O_3_.

**Figure 2:**
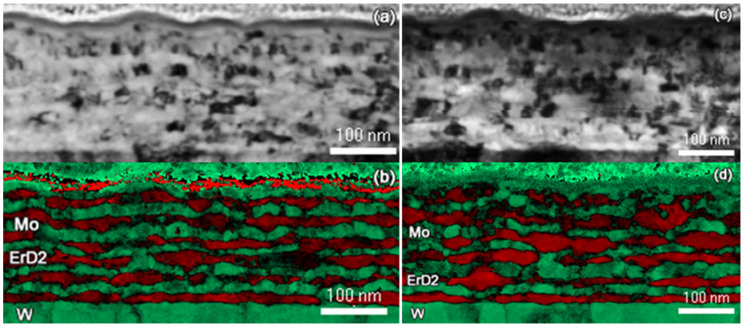
ACOM data of the as-deposited (**a**,**b**) and deuterated (**c**,**d**) Er/Mo multilayers. The top images (**a**,**c**) show the virtual BF image calculated from the ACOM data, while the bottom images (**b**,**d**) show the overlay of the index map, Mo and W in green and ErD_2_ in red.

The as-fabricated alternating Mo/Er layers are shown in Figure 1. The total composite film thickness is approximately 192 nm, with the average layer thicknesses of 14.4 nm (Er) and 14.7 nm (Mo). The corresponding oxygen EDXS map shows a correspondence between the elevated oxygen concentrations and Er layers, indicating the oxidation of the Er layers during and after fabrication. The oxide was likely formed during the dicing and loading processes. Erbium oxides were shown to form readily during the formation of Er films [43,44] and may impact the helium bubble nucleation and growth properties. ACOM data in Figure 2 shows the grain structure before and after hydriding. Mo and W both exhibit a bcc crystal structure with nearly identical lattice parameters and, thus, are indistinguishable in the data. Grains are elongated parallel to the surface and, therefore, are larger in size than the nanolayer thickness. The as-fabricated grain size, determined via ACOM, is a maximum of 31 nm (Er) and 33 nm (Mo). Mo and Er grains are expected to be near the same size after deposition. After hydriding, the ErD_2_ grain size increased to 62 nm, while the Mo grain size was unchanged. ErD_2_ grains are expected to be larger than the Er grains due to the phase transformation from hexagonal to fcc, which occurs during hydriding. Swelling accompanies this phase transformation as D is incorporated into the crystal structure. The total composite film thickness after hydriding is ~230 nm, corresponding to a 20% increase in total thickness. The Er layers swelled by 25% to an average thickness of 18.0 nm, exceeding the 10% expansion previously identified by others after hydriding Er to form ErD_2_; this was attributed to the larger volume expansion that occurred in Er_2_O_3_, thus providing further evidence for the inclusion of oxide in the Er/Mo layers [53,54]. 

### 3.2. Helium Bubble Nucleation

**Figure 3:**
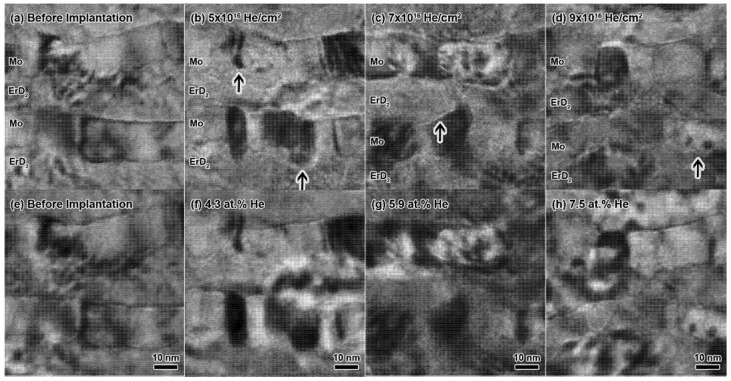
BF TEM images taken during the in-situ implantation at (**a**–**d**) −1 µm and (**e**–**h**) +1 µm defocus. Images were recorded 0° tilt in slightly different locations after several fluences. Bubbles are present along interfaces and within layers in (**b**/**f**), (**c**/**g**), and (**d**/**h**). Arrows indicate some examples of bubble nucleation at multilayer interfaces.

Spherical He bubbles nucleated both inside the nanolayers and at nanolayer interfaces after approximately 5 × 10^16^ He/cm^2^ during the in-situ ^4^He implantation. Figure 3a–h shows He bubble evolution as a function of fluence during in-situ implantation. Bubbles were observed to nucleate at the same time at interfaces and inside the nanolayers. It should be noted that bubbles are not easily identifiable in BF TEM until the diameter reaches approximately 1 nm. Bubbles appear to form elongated chains along interfaces, though these are difficult to decipher in the TEM images due to contrast effects at the interfaces and preferential milling at interfaces during the FIB sample preparation. Bubble sizes, as a function of region and fluence, are shown in Table 1. Though Table 1 does show some trends of bubble size increasing with fluence, particularly at the interfaces, there were no significant changes in bubble size within the layers throughout the experiment. Very little bubble growth is observed during in-situ implantation. Bubble density (Table 2) increases slightly with increasing fluence, showing that the multilayer geometry influenced the nucleation of an increased density of small bubbles without the growth of large matrix bubbles. No obvious denuded zones were identified along interfaces, but bubble size did appear notably larger at the interfaces, indicating that there was likely a denuded zone present at some scale.

### 3.3. Bubble Growth and Blistering at High Concentration

**Figure 4:**
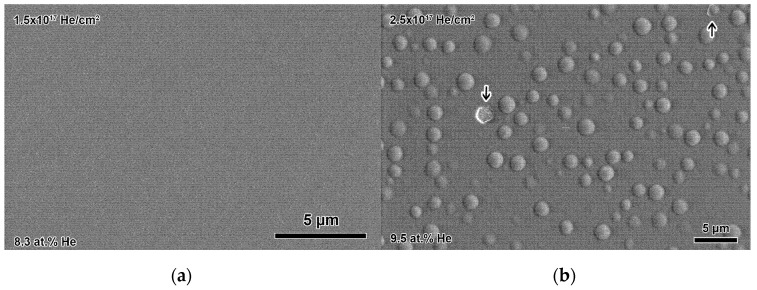
Secondary electron SEM images of bulk implanted multilayers, showing (**a**) the unchanged surface microstructure present up to a fluence of 1.5 × 10^17^ He/cm^2^ and (**b**) blisters on the surface after a fluence of 2.5 × 10^17^ He/cm^2^.

No changes in surface microstructure were observed after bulk ex-situ He implantation to fluences of up to 1.5 × 10^17^ He/cm^2^ (Figure 4a). In contrast, massive changes in surface topology were observed at 2.5 × 10^17^ He/cm^2^, where spherical dome-shaped blisters of 1.4 µm in diameter were scattered across the surface at a density of 0.06 blisters/µm^2^ (Figure 4b). In a few cases, blisters had burst, exposing a fractured surface, shown with an arrow in Figure 4b. Blisters were not observed with a peak implanted He concentration of 8.7 at.% (1.5 × 10^17^ He/cm^2^), but were observed after a peak implanted He concentration of 9.5 at.% (2.5 × 10^17^ He/cm^2^) was reached. There appears to be a critical concentration at which blister formation begins to occur.

**Figure 5:**
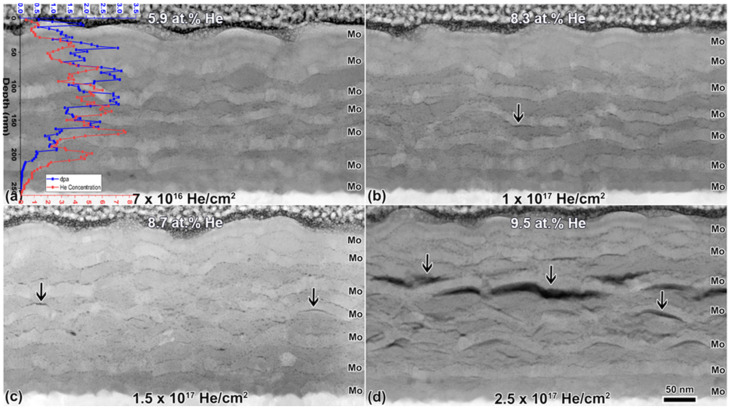
HAADF images of bulk implanted multilayers, showing He bubble microstructure as a function of fluence in (**a**–**d**) with SRIM He implant profile overlay in (**a**). He-filled bubbles and cracks appear darker than the matrix in the images and increase in size with increasing fluence. Arrows indicate signs of bubble coalescence and cracking along multilayer interfaces in (**b**,**c**). The image in (**d**) was taken in a region between blisters. Arrows in (**d**) indicate large nanocrack formation at multilayer interfaces. Bubbles appear dark due to the Z-contrast sensitivity of HAADF imaging.

Figure 5 shows cross-section HAADF images of the samples implanted with He ex-situ in bulk. He bubbles appear dark in these images, where the contrast is a function of *Z* to the power of *N*, and *N* is usually considered equal to 2 but can vary slightly [55,56]. In the cross-section images, the He bubble size and density are larger near the He concentration peak predicted by the SRIM profile. Bubbles are first visible in the TEM at around 3 at.% He in the sample implanted to 7 × 10^16^ He/cm^2^ (Figure 5a) and at around 3 at.% He in the sample implanted to 1 × 10^17^ He/cm^2^ (Figure 5b). In all images, bubbles appear to preferentially nucleate and grow along nanolayer interfaces. The arrows in Figure 5b–d show areas where the bubbles have grown together to form cracks along the nanolayer interfaces. These cracks become more apparent as the He concentration increases. At a fluence of 2.5 × 10^17^ He/cm^2^, large cracks are present at interfaces near the He concentration peak in the regions that do not intersect a blister (Figure 5d). Note that in some cases, particularly at the highest fluence of 2.5 × 10^17^ He/cm^2^, nanocracks do appear inside the layers. These nanocracks evolve similarly to those present at interfaces, from bubble chains forming within the layers. The largest crack formation always occurs at nanolayer interfaces near the He concentration peak. Bubble size and density data are provided in Table 3 and Table 4, respectively. Error bars are again large due to the difficulty in measuring small bubbles. Bubbles appear to remain near 1 nm in diameter in the ErD_2_ and Mo layers with no significant change as a function of fluence. The average bubble size at interfaces presents a more distinct increasing trend, though all values are still the same within error. Bubble size becomes more difficult to define and measure as bubbles form chains and nanocracks at higher fluences. Bubble density within the ErD_2_ and Mo layers decreases as the fluence increases. This is attributed to bubble coalescence and growth into larger bubbles, chains, or nanocracks.

**Figure 6:**
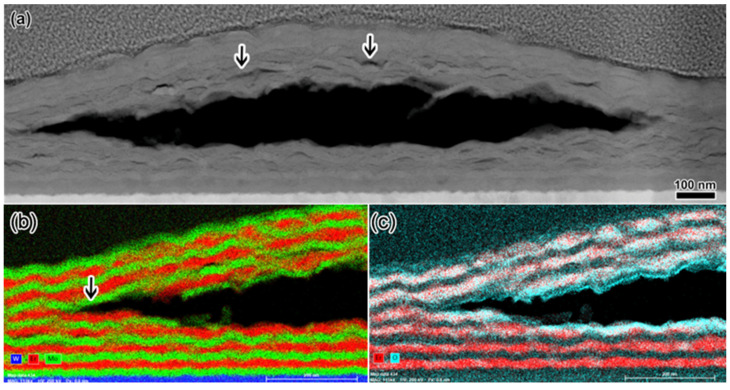
Blister formation in bulk implanted multilayers after the highest implantation fluence of 2.5 × 10^17^ He/cm^2^. (**a**) HAADF image of a blister cross-section. Arrows point to crack formation along multilayer interfaces. The gas-filled blister gap is apparent in (**a**). Frame (**b**) shows EDXS maps of the Mo (green) and Er (red) layers. Arrow points to the fracture surface where the blister gap formed. The O EDXS map (blue) in (**c**) allows identification of the layers (primarily Er layers) where oxide is present.

Blister formation appears to occur at a concentration when the cracks along the nanolayer interfaces become large enough to connect. This is not observed after 1.5 × 10^17^ He/cm^2^ (8.7 at.% peak He, Figure 4a and Figure 5c) but is observed after 2.5 × 10^17^ He/cm^2^ (9.5 at.% peak He, Figure 5d). Figure 6a shows a representative blister cross-section in the same TEM sample shown in Figure 5d, which was prepared from the sample implanted to 2.5 × 10^17^ He/cm^2^. The blisters are, on average, 1.4 µm in diameter, so any given TEM sample will likely contain blister cross-sections as well as areas without blisters. The blister cross-section shows a large gap, which was presumably formed during He gas building inside the region. The blister gap formed at the same depth where the large nanocracks were present at nanolayer interfaces in Figure 5d, at the He concentration peak. Small nanocracks still appear at the nanolayer interfaces below and above the blister gap, some of which are indicated by arrows in Figure 6a. The EDXS maps in Figure 6b show where the ErD_2_ and Mo layers are with respect to the blister gap. Fracture appears to occur primarily between Mo and ErD_2_ layers, but then jogs through the layers toward the edges of the blister (shown with an arrow in Figure 6b), likely along a grain boundary containing small He clusters or He bubbles. Figure 6c shows that most oxide exists in the ErD_2_ layers, and that oxide exists both above and below the blister gap.

## 4. Discussion

To the best of our knowledge, this represents the first work exploring He bubble behavior in metal hydride multilayers, complementing the prior work on metal multilayers and pure metal hydrides. In comparison to ErD_2_ samples without the presence of multilayers [57], 1.8 nm diameter, inter-granular spherical He bubbles are observed at a He fluence of 2 × 10^17^ ions/cm^2^ (5.5 at.% He). The average bubble density is 2 × 10^12^ cm^−2^. The average size is larger, but density lower than in the multilayer samples exposed to a similar He fluence studied here. Bubble chains and nanocracks are observed after 5 × 10^17^ ions/cm^2^ fluence irradiation, initiating at a He concentration of approximately 7.5 at.%, compared to the 9 at.% observed in the multilayered samples in Figure 5. This may indicate that the multilayer samples can tolerate more He due to the presence of the Er/Mo interfaces, similar to prior work on Cu/Nb multilayer systems [26,27]. 

In prior work on multilayers, fcc/bcc interfaces were shown to readily trap He, both in experiments and in modelling, to limit He bubble growth within the layers [26]. However, in our study, bubbles in the layers and at interfaces appear to form at the same He fluences. As the fluence progresses, the bubbles grow more rapidly within the interfaces, indicating some preferential absorption of He at the interfaces. The diminished ability to capture He at the incubation stages of bubble formation may be due to the oxide that developed to form erbium oxide. We desired to form fcc/bcc interfaces with Mo/ErD_2_. However, EDXS shows that we have a significant percentage of Mo/Er_2_O_3_ interfaces compared to that which is desired fcc/bcc interfaces. These new interfaces might act as less ideal interfaces for absorbing He. Thus, due to the oxidation that likely occurred during the hydriding step of the process, the He sequestration effects of the multilayers is diminished. However, He trapping at metal–metal oxide interfaces was shown to be an effective way to manage He inventory, such as in ODS [58,59,60]. DFT simulations showed the propensity for He to bind to Er oxide [61]. Thus, more study is needed to investigate the impact of the oxide on He bubble nucleation and growth in Er systems.

At low fluence, the uniform bubble densities may be due to the room temperature nature of experiments. The formation of homogenous bubbles at room temperature He implantation was seen in other material systems and is due to the lack of diffusional energy that would promote He migration and large bubble formation through Oswald Ripening or Migration and Coalescence [62]. As the fluence progresses, the bubbles link to form chains and, ultimately, the accumulation of He at the interfaces may have caused the decohesion, or weakened the interface, allowing for the rupture there. He accumulation at the interfaces can lead to decohesion. Beginning at a fluence of 1.5 × 10^17^ ions/cm^2^, nanocracks form at the interfaces with a length of approximately 20 nm. However, this He accumulation leads to lateral bubble connection, which leads to cracking, likely because the nanocrack is growing along the region of least resistance along the interface. The pressure increases such that the crack size is larger than the critical crack size, resulting in fracturing of the matrix.

Blister formation and growth is commonly attributed to bubble accumulation, an increase in the He gas pressure, and overcoming of the strength of the material; in this case, it would be due to overcoming of the interfacial strength of the layers. Blistering can be expected under high energy irradiation where the He accumulation is well beneath the surface, limiting the ability to have surface release. The linkage of bubbles along the interfaces allowed for He gas pressure to increase to exert a force greater than the interfacial strength of the interface. The interfaces between layers are the weak point in the matrix, and the He accumulation caused debonding of the layers. Despite the ability for multilayer structures to accommodate He damage, they may be preferential delamination and failure points. Studies under high temperature annealing following He implantation show delamination and blister development at layer interfaces in Cu/Nb multilayers [36]. Thus, the growth of interface bubbles under annealing shows the weakness of these interfaces once loaded with He and that expanding He bubbles can cause delamination. Blistering at a critical dose without a temperature influence was observed in other materials [62]. Thus, due to the high energy implanting well beneath the surface and the buildup of He due to the continued He implantation at high fluence, blistering occurs.

The development of cracks in the blister lid and below the blister as well as those that form the blister seem to support the interbubble fracture model [17,18,19,63], and was also seen in He-implanted W [16]. The blister base develops from the region of peak He concentration. The He bubbles coalesce to form cracks, and their lateral accumulation and coalescence forms the cavity under high He fluence. In the lid and beneath the blister, the accumulated He concentration results in nanocracks, yet is not yet high enough to induce sufficient linkage and cause blistering. 

For in-situ TEM irradiations, one must also discuss the thin film effect of performing an irradiation of a 100 nm-thick sample. In these types of experiments, the increased free surface area can alter the defect dynamics by promoting annihilation at the surfaces more rapidly and efficiently than would occur in a bulk sample. However, in this case of irradiation of 20 nm-thick multilayers, this should not have a significant impact because the sample thickness is greater than the multilayer thickness. Thus, the He introduced to the sample will have a shorter diffusion pathway to—and a greater likelihood of—interaction with a multilayer interface than the free surface. 

Metal hydrides utilized in complex nuclear environments could be subject to combined H and He isotope exposure, depending on the application, and must tolerate these conditions and maintain structural stability. This work examined tailoring interfaces for controlling He behavior in nanolayered composites containing metal hydrides. We show that introducing microstructural features, such as interfaces, can enhance stable He storage in the presence of H isotopes. This work establishes a pathway for future studies tailoring candidate hydrogen material for enhanced stability through microstructure modifications such as second phase inclusions, twin boundary introductions, or controlled porosity introductions, and subsequent in-situ and ex-situ investigations under combined H and He exposure conditions. 

## 5. Conclusions

Multilayered metal/metal hydride composites demonstrate enhanced radiation tolerance under He irradiation, altering the He bubble distributions by increasing the density of interfaces. This work examined He bubble nucleation and growth in deuterated Er/Mo multilayer systems. ErD_2_/Mo multilayers were fabricated via magnetron sputtering, and subsequently deuterated, resulting in swelling of the multilayers. Early-stage He bubble formation was subsequently studied with in-situ TEM under 10 keV He bombardment, while late-stage He bubble linkage and blister formation was studied with ex-situ 30 keV He implantations. Under low fluence He irradiation, homogenous bubble nucleation of approximately 1 nm diameter bubbles is observed within Er/Mo layers and approximately 2 nm diameter bubbles at the interfaces, while the bubbles are not observed to grow in size, but the density increases slightly as the fluence increases to 9 × 10^16^ cm^−2^ fluence. High fluence, ex-situ irradiations show the growth of bubbles as fluence increases within interfaces, mainly due to bubble linkage at high fluence. As the fluence increases to 2.5 × 10^17^ cm^−2^, blistering of the specimen surface is observed due to the linkage of bubbles at layer interfaces and the build-up of He gas pressure. This work suggests that, similar to metal multilayers, metal hydride multilayered samples can also tolerate He inclusion to limit material degradation. 

## Figures and Tables

**Table 1:** He bubble size (diameter) measured during in-situ He implantation, as a function of fluence.

He Fluence (cm^−2^)	ErD_2_ Layers (nm)	Mo Layers (nm)	Interfaces (nm)
5 × 10^16^	0.8 ± 0.2	0.9 ± 0.2	1.9 ± 1.0
7 × 10^16^	0.9 ± 0.2	1.0 ± 0.2	2.1 ± 1.0
9 × 10^16^	1.1 ± 0.2	1.0 ± 0.2	2.7 ± 2.2

**Table 2:** He bubble density measured during in-situ He implantation, as a function of fluence.

He Fluence (cm^−2^)	ErD_2_ Layers (cm^−2^)	Mo Layers (cm^−2^)
5 × 10^16^	3.6 ± 1.3 × 10^12^	4.1 ± 1.0 × 10^12^
7 × 10^16^	6.7 ± 3.6 × 10^12^	5.3 ± 2.6 × 10^12^
9 × 10^16^	7.6 ± 0.61 × 10^12^	7.4 ± 1.0 × 10^12^

**Table 3:** Average He bubble diameter at each ex-situ He implantation fluence.

	Average Bubble Diameter (nm)			
**He fluence (cm^−2^)**	7 × 10^16^	1 × 10^17^	1.5 × 10^17^	2.5 × 10^17^
**ErD_2_**	1.6 ± 0.5	1.4 ± 0.4	1.5 ± 0.5	1.7 ± 0.4
**Mo**	1.7 ± 0.4	1.5 ± 0.3	1.6 ± 0.4	1.6 ± 0.4
**Interfaces**	1.4 ± 0.3	1.6 ± 0.4	2.0 ± 0.5	2.9 ± 0.9

**Table 4:** He bubble density at each ex-situ He implantation fluence.

	Bubble Density (cm^−2^)			
**He fluence (cm^−2^)**	7 × 10^16^	1 × 10^17^	1.5 × 10^17^	2.5 × 10^17^
**ErD_2_**	2.1 × 10^12^	1.0 × 10^12^	0.8 × 10^12^	0.7 × 10^12^
**Mo**	1.9 × 10^12^	1.3 × 10^12^	1.2 × 10^12^	0.8 × 10^12^
**Interfaces**	n/a	n/a	n/a	n/a

## Data Availability

Not applicable.

## Supplementary Materials

The following are available online at https://www.mdpi.com/article/10.3390/ma14185393/s1, Figure S1: Grazing-incidence XRD showing the presence of oxide in implanted multilayered films.
