# Supplementary material for: Helium Bubbles and Blistering in a Nanolayered Metal/Hydride Composite
Source: Materials (Basel). 2021 Sep 18;14(18):5393. doi: 10.3390/ma14185393 (PMC8471048; doi:10.3390/ma14185393)
Supplement: Supplementary file 1 [file materials-14-05393-s001.zip › materials-1367905-supplementary.pdf]

coatings

MDPI

Article

Helium Bubbles and Blistering in a Nanolayered Metal/Hydride Composite

Caitlin A. Taylor [1,2], Eric Lang [3], Paul G. Kotula [3], Ronald Goeke [2], Clark S. Snow [2], Yongqiang Wang [1] and Khalid Hattar [3,*]

[1] Materials Science and Technology Division, Los Alamos National Laboratory, Los Alamos, NM 87545, USA; caitlin@lanl.gov (C.A.T.); yqwang@lanl.gov (Y.W.)
[2] Component Science, Engineering, and Production Center, Sandia National Laboratories, Albuquerque, NM 87185, USA; rsgoeke@sandia.gov (R.G.K.); cssnow@sandia.gov (C.S.S.)
[3] Material, Physical, and Chemical Sciences Center, Sandia National Laboratories, Albuquerque, NM 87185, USA; ejlang@sandia.gov (E.L.); pgkotul@sandia.gov (P.G.K.); khattar@sandia.gov (K.H.)
* Correspondence: khattar@sandia.gov

Citation: Taylor, C.A.; Lang, E.; Kotula, P.G.; Goeke, R.; Snow, C.; Wang, Y. Helium Bubbles and Blistering in a Nanolayered Metal/Hydride Composite. *Materials* **2021**, *14*, 5393. https://doi.org/10.3390/ma14185393

Received: 19 August 2021
Accepted: 13 September 2021
Published: 18 September 2021

Publisher's Note: MDPI stays neutral with regard to jurisdictional claims in published maps and institutional affiliations.

Copyright: © 2021 by the authors. Submitted for possible open access publication under the terms and conditions of the Creative Commons Attribution (CC BY) license (https://creativecommons.org/licenses/by/4.0/).

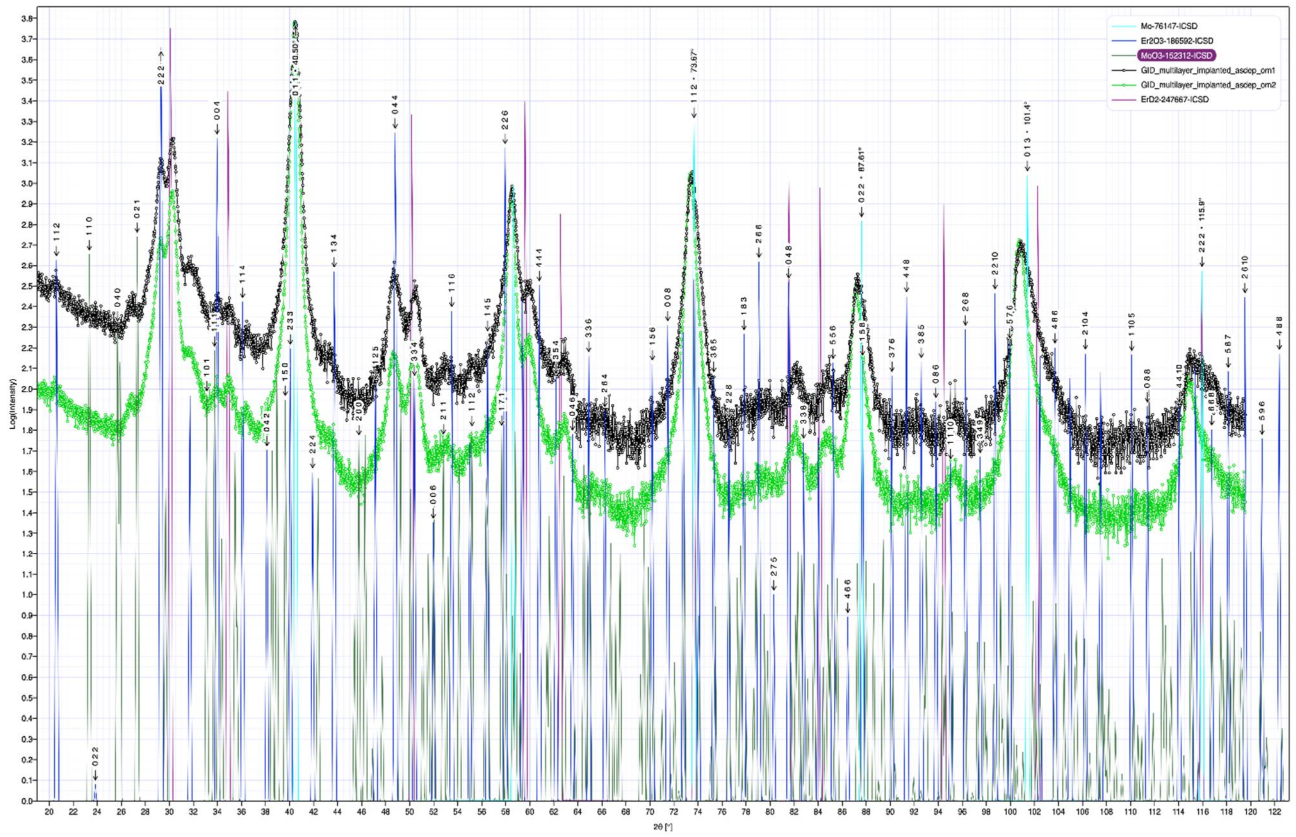

Figure S1. Grazing-incidence XRD showing the presence of oxide in the multilayered films after hydriding.
